# Supplementary material for: Electrochemical measurements at human iPSC-derived FOXA2 dopaminergic neurons suggest a role for partial release in presynaptic plasticity
Source: QRB Discov. 2026 Mar 23;7:e3. doi: 10.1017/qrd.2026.10019 (PMC13125770; doi:10.1017/qrd.2026.10019)
Supplement: Gu et al. supplementary material [file S2633289226100192sup001.pdf]

Electrochemical Measurements at Human iPSC-Derived FOXA2 Dopaminergic Neurons Suggest a Role for Partial Release in Presynaptic Plasticity

Chaoyi Gu, Alicia A. Lork, Soodabeh Majdi, Stefania Rabasco, Huashan Peng, Anjie Ni, Carl Ernst, Andrew G. Ewing*

* To whom correspondence may be addressed. Email: andrewe@chem.gu.se

Supplementary Materials and Methods

Chemicals and solutions

All chemicals were purchased from Merck (Sweden) and of analytical grade, unless otherwise stated. Isotonic solution was used during electrochemical measurements and calcium imaging, and consisted of 150 mM NaCl, 5 mM KCl, 1.2 mM $MgCl_2$, 2 mM $CaCl_2$, 5 mM glucose, and 10 mM HEPES. Exocytosis was stimulated with 55 mM NaCl, 100 mM KCl, 1.2 mM $MgCl_2$, 2 mM $CaCl_2$, 5 mM glucose, and 10 mM HEPES (stimulation solution). The pH of all solutions was adjusted to 7.4 with 3 M NaOH and all solutions were filtered with 0.45 μm filters (VWR, Sweden) before use.

Plasmid constructs

To generate the FOXA2-ntdTomato reporter line, two vectors were used: a CRISPR-cas9 vector encoding the gRNA targeting the FOXA2 stop codon, and a donor vector carrying the tdTomato-reporter cassette flanked by homology arms.

The CRISPR-Cas9 construct targeting the stop codon of FOXA2 was designed using the CRISPick software, yielding the guide RNA sequence 5′-GAAGCCGTCGTCTTCTTAAG-3′. This sequence was synthesized by GenScript (USA) and cloned into the pX459-HypaCas9-mR2-AAVS1_sgRNA vector (Addgene plasmid #183890).

The donor template vector was constructed by synthesizing the left (910 bp) and right (819 bp) homology arms flanking the FOXA2 stop codon. These homology arms were inserted into the pUC19-SOX2-T2A-2xNLS-tdTomato-F2A-Puro backbone using the restriction enzymes SbfI, NheI, AscI, and NotI, respectively.

Generation of FOXA2-ntdTomato reporter iPSC line

The FOXA2-ntdTomato reporter iPSC line was generated by first transfecting control dopaminergic neural progenitor cells (NPCs) followed by reprogramming the NPCs to iPSCs. Control dopaminergic NPCs were co-transfected with the FOXA2-targeting CRISPR-Cas9 vector along with the donor plasmid pUC19-FOXA2-T2A-2xNLS-tdTomato-F2A-Puro. Transfected cells were selected using 1 μg/mL puromycin (ThermoFisher) in dopaminergic NPC expansion medium for 15–21 days.

tdTomato-positive cells were then isolated and reprogrammed into iPSCs using the pCXLE episomal vector toolkit (Addgene plasmids #27078, #27080, and #27077). iPSC colonies were selected and expanded 15–21 days post-reprogramming.

Cell culture

FOXA2 NPCs were thawed and cultured in a low-attachment petri dish (Fisher Scientific, Sweden) for 2-3 days until neural progenitor aggregates with the size of 100-200 μm were formed in the suspension. The NPC culture medium was made of STEMDiff Neural Progenitor Basal Medium (Stemcell Technologies, Canada) supplemented with 200 ng/mL Sonic Hedgehog (Genscript, USA). 10 μM Y-27632 ROCK inhibitor (Stemcell Technologies, Canada) was added to the medium at the beginning of the culture to assist the survival of the FOXA2 NPCs. The suspension was then filtered with a 40 μm cell strainer (Fisher Scientific, Sweden) to collect the aggregates and remove single cells. The strainer was subsequently washed with the culture medium and the solution was collected into a T25 culture flask (Nunc™ EasYFlask™, Fisher Scientific, Sweden) coated with

poly-D-lysine (PDL) and laminin. The culture was kept in an incubator at 37 °C and 5% CO_2. Medium was changed every two days and the cells were passaged once confluence was reached.

After 1-2 passages, NPCs were seeded on 35-mm dishes (Nunc™ EasYFlask™, Fisher Scientific, Sweden) coated with PDL and laminin. Cells were cultured in the NPC culture medium until 70-80 % confluence and then the medium was replaced completely by differentiation medium. The composition of the differentiation medium included BrainPhys (Stemcell Technologies, Canada), 2 % B27 (Gibco, Fisher Scientific, Sweden), 1 % N2 (Gibco, Fisher Scientific, Sweden), 20 ng/mL BDNF (Genscript, USA), 20 ng/mL GDNF (Genscript, USA), 200 nM ascorbic acid (Stemcell Technologies, Canada), 1 mM dibutyryl cAMP (Stemcell Technologies, Canada), and 1 µg/mL laminin. Half medium change was performed every 2-3 days during a total of 2 weeks. For cells differentiated with BAY-K8644, 2 µM BAY-K8644 was added to the differentiation medium at the beginning and medium containing 4 µM BAY-K8644 was used for half-medium change throughout the differentiation period (1).

Fabrication of electrodes

Nanotip electrodes were fabricated according to a protocol published previously (2). A 5 µm carbon fiber was aspirated into a glass capillary (borosilicate, O.D.: 1.2 mm, I.D.: 0. 69 mm, 10 cm length, Sutter Instruments, USA). The capillary was subsequently pulled into two parts with a vertical pipette puller (model PE-21, Narishige, Inc., Japan) and the carbon fiber outside the glass was cut to 50-100 µm length. The fiber was then flame-etched with an ethanol lamp (VWR, Sweden) to form a sharp tip and afterwards, the electrodes were sealed by dipping into epoxy (G A Lindberg ChemTech AB, Sweden) for 1.5 min and washed shortly in acetone (5-10 s). In the end, the electrodes were baked in an oven at 100 °C overnight to cure the epoxy. The electrodes were tested in 100 µM dopamine solution with cyclic voltammetry (-0.2 V to +0.8 V vs. Ag/AgCl, scan rate: 100 mV/s) right before the electrochemical measurements. Electrodes showing stable steady-state currents were used for the measurements.

Open carbon nanopipettes (CNPs) were made as previously described (3). Briefly, a glass capillary (quartz, O.D.: 1.0 mm, I.D.: 0. 70 mm, Sutter Instruments, USA) was first pulled into two parts with a laser pipette puller (P-2000, Sutter Instruments, USA). The pipettes were then placed approximately in the center of a furnace (Carbolite Gero, UK), and the furnace was flushed with argon gas (Instrument Argon 5.0, Linde, Sweden) and heated until 980 °C. Methane gas (Chemical Methane 2.5, Linde, Sweden) was turned on and the mixture gas of argon and methane was passed through the furnace for 30 min to deposit carbon onto the inner surface of the pipettes. Afterwards, methane was turned off and the furnace was allowed to cool down under the continuous flow of argon.

Electrochemical measurements and data analysis

Differentiation medium was removed from the dish before the electrochemical experiments and cells were washed gently with isotonic solution for 2 times. Cells were bathed in 2 mL isotonic solution and kept at 37 °C on a heating plate during the entire measurement. Each dish was measured for maximum 40 min to ensure optimal condition for the cells. Both SCA and IVIEC were carried out on an inverted microscope (IX73 or IX81, Olympus) which was placed inside a Faraday cage. A potential of +700 mV versus an Ag/AgCl reference electrode was applied on the electrode using an Axopatch 200B potentiostat (Molecular Devices, USA) to oxidize dopamine molecules. For SCA, an electrode with a conical-shaped tip was positioned either on top of a bouton or between axons of two neurons. Exocytosis was stimulated using a glass pipette filled with stimulation solution which was coupled to a microinjection device (Picospritzer II, General Valve Corporation,

USA). The pipette was placed around 20 µm away from the electrode position, and the pressure and the duration of the stimulation were 20 psi and 10 s, respectively. For IVIEC, a CNP was used to pierce into either the cell body area or a bouton of a neuron and remained inside untill the end of the recording. Sampling rate for both SCA and IVIEC was 20 kHz, and signal outputs were filtered and digitized at 2 and 5 kHz, respectively.

A script written by the David Sulzer group (Columbia University) was used in Igor Pro 6.37 software to analyze all electrochemical data (4). A 1 kHz Binomial sm. filter was applied to the data and the threshold for spike detection was set to be 5 times the standard deviation of the noise. All spikes selected by the software were checked to avoid false positives, and spikes that were undetected by the software were checked and manually added. To calculate number of molecules from individual spikes, Faraday's law $N = Q/nF$ was applied. Q is the charge of the spike and is obtained by integrating the area under the spike, n is the number of electrons transferred in the electrochemical oxidation reaction with 2 electrons for the oxidation of a dopamine molecule, and F is the Faraday constant which is 96,485 C/mol. Means of medians from single cells were used to calculate average number of molecules and average $t_{1/2}$. Bar graphs, histograms, and statistics were done in GraphPad Prism 5. Pairs of data sets were compared with Mann-Whitney test, *: $p < 0.05$, **: $p < 0.01$.

Calcium imaging and data analysis

For calcium imaging experiments, NPCs were seeded on µ-slide 4 or 8 well glass bottom chamber slide (Ibidi GmbH, Germany) coated with PDL and laminin, and differentiated for 2 weeks. Prior to the experiment, cells were washed with isotonic solution for 2 times and incubated with isotonic solution containing 1 µM fluo-4 calcium indicator for 30 min (1). Afterwards, cells were washed again with isotonic solution to remove the fluorescence dye and kept in isotonic solution during the experiment. A Zeiss LSM 700 laser scanning microscopy (Centre for Cellular Imaging, Sahlgrenska Academy, University of Gothenburg, Sweden) equipped with a Plan-Apochromat 20x objective (numerical aperture (NA) = 0.8) was used to perform calcium imaging. Fluo-4 was excited at the wavelength of 488 nm and emission signals over 500 nm were collected every 484.07 ms. After a baseline of 15 s, 100 mM K^+ stimulation solution was injected to initiate exocytosis and in total, 90 s signals were recorded. Images were acquired with Zen microscope software (Zeiss) and different areas along the neuronal axons were selected as regions of interest to perform the analysis.

Calcium imaging data were divided into a time interval of 15 s for the first 60 s. Average fluorescence intensity from each cell for each time interval was first calculated, then average intensity from all cells within one group for each time interval was obtained. Average fluorescence intensity within the first 15 s was considered as the baseline intensity and was used to calculate relative intensity fluctuations upon stimulation for exocytosis.

Sample preparation and TEM imaging

For TEM imaging, NPCs were plated on glass bottom dishes (MatTek Life Sciences, USA) coated with PDL and laminin, with or without subsequent differentiation. Cells were then washed with DPBS and fixed with Karnovsky fixative in 0.1 M sodium cacodylate buffer for 30 min, followed by washing with 150 mM sodium cacodylate buffer and kept overnight at 4 °C. Post-fixation with 1% osmium tetroxide at 4 °C for 30 min was performed, and 1% uranyl acetate was applied for 20 min for additional contrast. A series of dehydration steps was afterwards carried out with increased concentration of ethanol and the cells were then embedded in Agar100 resin. Resin-filled capsules were loaded onto the cell monolayers and polymerized at 60 °C for approximately 16 h. The

embedded samples were sectioned into 150 nm thick sections and placed onto copper finder grids (Electron Microscopy Sciences, FCF200F1-CU).

TEM imaging was performed using a Talos L120C TEM microscope (Thermo Scientific) operated at 120 keV. Images were acquired at 11000x magnification.

Immunostaining and confocal imaging

NPCs were seeded on coverslips coated with PDL and laminin in a 24-well plate (Fisher Scientific, Sweden). After differentiation for 2 weeks, cell medium was removed and cells were washed 3 times with DPBS, and fixed in paraformaldehyde solution (4% in PBS, Fisher Scientific, Sweden) for 30 min. Afterwards, cells were washed 3 times with PBS and permeabilized in 0.1% Triton X in PBS for 15 min, which was followed by washing 3 times with PBS. 1% bovine serum albumin in PBS and 0.1% Tween were then used for blocking for 1 h, and primary antibodies (rabbit anti-beta III tubulin, mouse anti-TH, Abcam) were incubated in the blocking solution for another 1 h and washed 3 times with PBS. Secondary antibodies (STAR 580 anti-rabbit, STAR 635 anti-mouse, Abberior) were subsequently incubated in the blocking solution for 1 h, followed by 2 times wash with PBS. Finally, cells were embedded and imaged on a confocal microscope (Abberior, Germany). STAR 580 was imaged with excitation at 580 nm and detection at 600-620 nm. STAR 635 was imaged with excitation at 640 nm and detection at 650-720 nm.

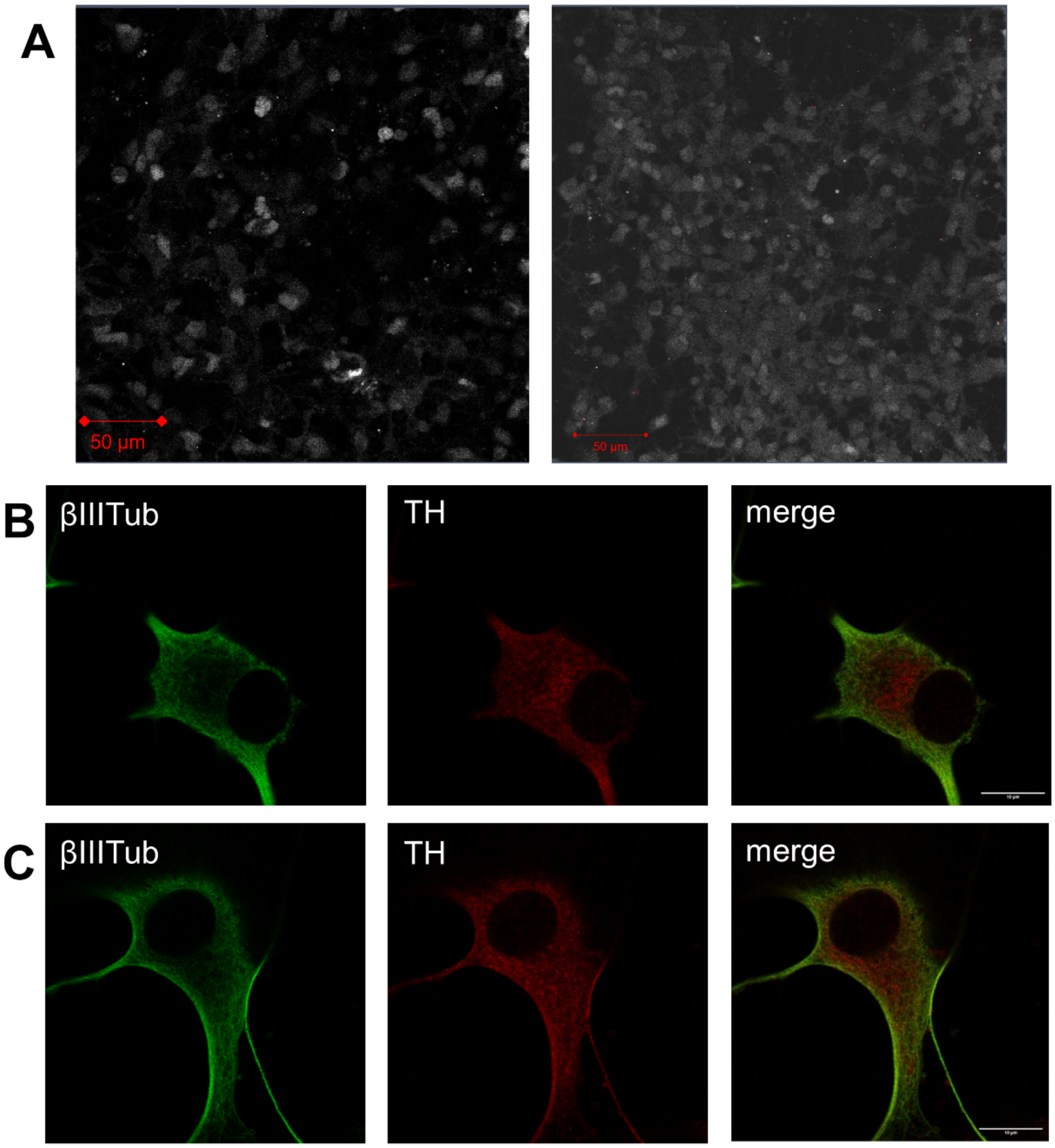

Fig. S1. (A) Representative fluorescence images showing FOXA2 expression level in differentiated DA neurons. Scale bars: 50 µm. (B-C) Immunostaining of beta 3 tubulin (green) and TH (red) expressions in differentiated FOXA2 DA neurons. Scale bars: 10 µm.

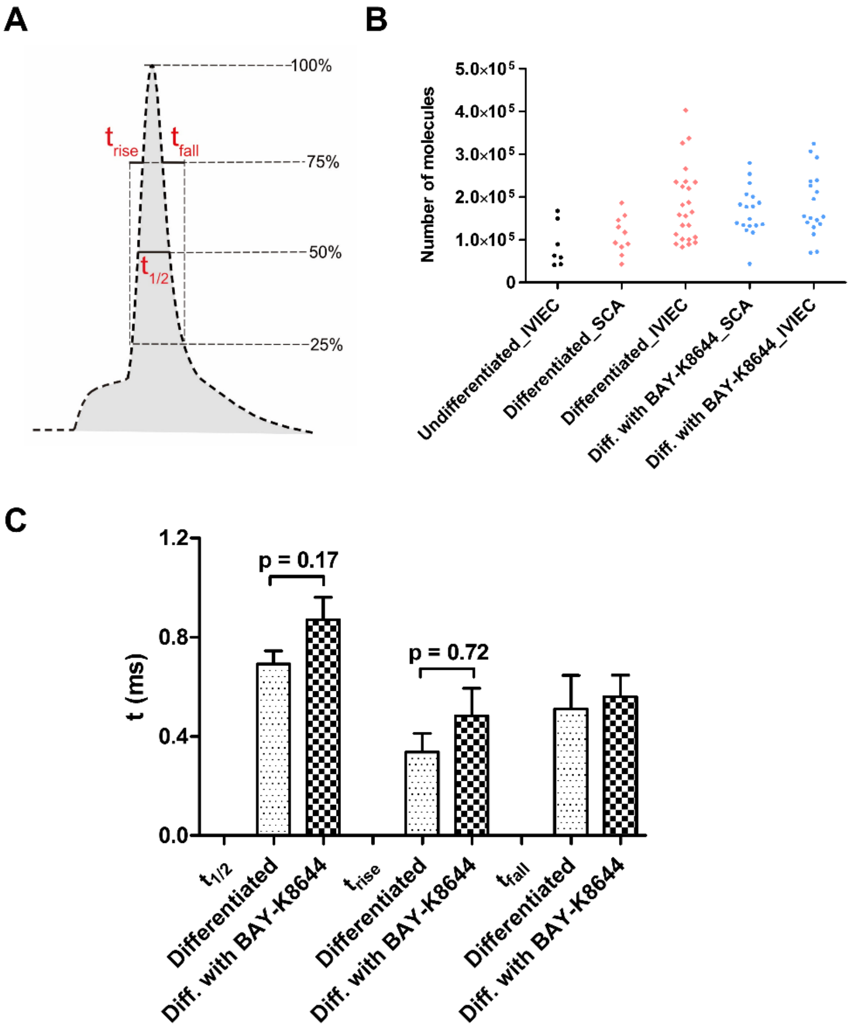

Fig. S2. (A) Scheme illustrating the shape of an amperometric spike. Area under the spike is shown in grey and parameters $t_{1/2}$ (half width of the spike), t_{rise} (time taken to rise from 25 to 75% of the spike height), and t_{fall} (time taken to fall from 75 to 25% of the spike height) are depicted in the scheme. (B) Scatter dot plot showing distributions of number of molecules from all cells under different conditions, including NPCs (undifferentiated), differentiated DA neurons, and DA neurons differentiated with BAY-K8644. DA release and vesicular DA content were quantified by SCA and IVIEC, respectively. 7 cells were counted for IVIEC from NPCs and over 10 cells were counted for the rest of the groups. (C) Comparison of average $t_{1/2}$, t_{rise}, and t_{fall} measured by SCA between DA neurons differentiated without or with BAY-K8644. More than 10 cells were counted for each group. Error bars represent means of medians ± SEM. Data sets were compared with Mann-Whitney t test and p values are shown in the graph.

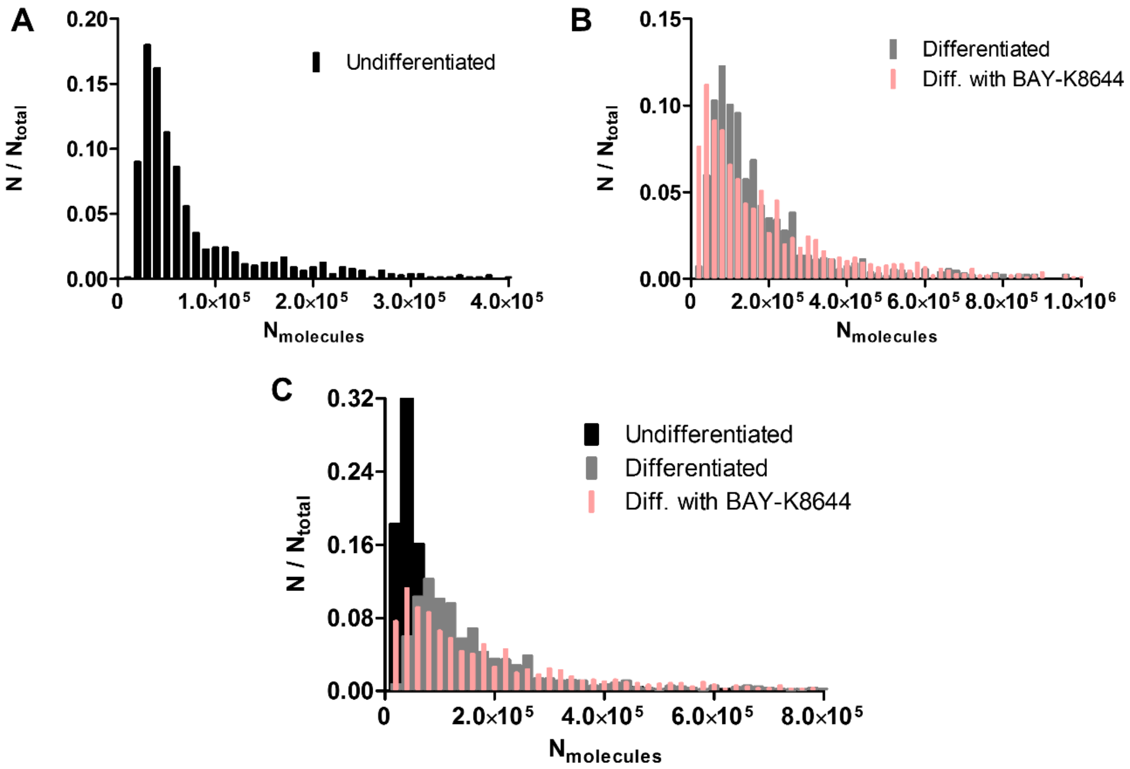

Figure S3. (A) Normalized frequency histogram of the distribution of number of DA molecules quantified by IVIEC from NPCs (undifferentiated, 790 events from 7 cells), bin size = 1x10^4. (B) Normalized frequency histograms of the distributions of number of DA molecules quantified by IVIEC from differentiated DA neurons without (1256 events from 25 cells) or with BAY-K8644 (1063 events from 18 cells), bin size = 2x10^4. (C) Normalized frequency histograms of the distributions of number of DA molecules quantified by IVIEC from all three groups, bin size = 2x10^4.

SI References

1. Jefri M*, et al.* (2020) Stimulation of L-type calcium channels increases tyrosine hydroxylase and dopamine in ventral midbrain cells induced from somatic cells. *Stem Cells Transl Med* 9(6):697-712.
2. Li X, Majdi S, Dunevall J, Fathali H, & Ewing AG (2015) Quantitative measurement of transmitters in individual vesicles in the cytoplasm of single cells with nanotip electrodes. *Angew Chem Int Ed* 54(41):11978-11982.
3. Hu K*, et al.* (2014) Open Carbon Nanopipettes as Resistive-Pulse Sensors, Rectification Sensors, and Electrochemical Nanoprobes. *Analytical Chemistry* 86(18):8897-8901.
4. Mosharov EV (2008) Analysis of single-vesicle exocytotic events recorded by amperometry. *Methods Mol Biol* 440:315-327.
